# Discrimination of Etiologically Different Cholestasis by Modeling Proteomics Datasets

**DOI:** 10.3390/ijms25073684

**Published:** 2024-03-26

**Authors:** Laura Guerrero, Jorge Vindel-Alfageme, Loreto Hierro, Luiz Stark, David Vicent, Carlos Óscar S. Sorzano, Fernando J. Corrales

**Affiliations:** 1 Centro Nacional de Biotecnología (CNB-CSIC), c/Darwin, 3, 28049 Madrid, Spain; lauguegonz@gmail.com (L.G.); jorge.vindel@cnb.csic.es (J.V.-A.); coss@cnb.csic.es (C.Ó.S.S.); 2 IdiPAZ, Instituto de Investigación Sanitaria (Health Research Institute), Hospital Universitario La Paz, Paseo de la Castellana 261, 28046 Madrid, Spain; marial.hierro@salud.madrid.org (L.H.); luiz.stark@salud.madrid.org (L.S.); david.vicent@salud.madrid.org (D.V.)

**Keywords:** liver, cholestasis, quantitative proteomics, machine learning

## Abstract

Cholestasis is characterized by disrupted bile flow from the liver to the small intestine. Although etiologically different cholestasis displays similar symptoms, diverse factors can contribute to the progression of the disease and determine the appropriate therapeutic option. Therefore, stratifying cholestatic patients is essential for the development of tailor-made treatment strategies. Here, we have analyzed the liver proteome from cholestatic patients of different etiology. In total, 7161 proteins were identified and quantified, of which 263 were differentially expressed between control and cholestasis groups. These differential proteins point to deregulated cellular processes that explain part of the molecular framework of cholestasis progression. However, the clustering of different cholestasis types was limited. Therefore, a machine learning pipeline was designed to identify a panel of 20 differential proteins that segregate different cholestasis groups with high accuracy and sensitivity. In summary, proteomics combined with machine learning algorithms provides valuable insights into the molecular mechanisms of cholestasis progression and a panel of proteins to discriminate across different types of cholestasis. This strategy may prove useful in developing precision medicine approaches for patient care.

## 1. Introduction

Cholestatic disease is caused by the disruption of bile flow from the hepatocyte to the small intestine [1] Bile constitutes the primary way for the elimination of cholesterol excess, bilirubin, and non-water-soluble xenobiotics that cannot be excreted through urine. Under physiological conditions, bile is produced by the hepatocytes and secreted to the bile canaliculus, then it flows to the gallbladder, where it is stored until it is released into the duodenum. Both the defect of bile formation and secretion and the block of bile flow can lead to cholestasis, either intrahepatic or extrahepatic, regardless of where the bile accumulation occurs. Cholestasis can also be classified as obstructive or non-obstructive according to the reason for the bile flow disruption. The main alterations observed in cholestasis include jaundice, pruritus, bilirubinemia, elevated serum bile salts, and alkaline phosphatase [2] The etiology of cholestasis is diverse, including cholestasis of pregnancy, drug-induced cholestasis, inflammatory cholestasis, or genetic defects leading to different types of familial cholestasis.

Progressive familial intrahepatic cholestasis (PFIC) is a severe rare disease presented in 1/10,000 to 1/20,000 births [3] PFIC can be classified into subtypes according to the affected gene. The impairment of the encoded proteins involved in the transport of bile acids or phospholipids, such as phosphatidylserine, phosphatidylethanolamine, or phosphatidylcholine, is ultimately the triggering factor of PFIC. PFIC1 results from a defect in the ATP8B1 gene encoding for the FIC1 protein. PFIC2 is caused by the deficit of the ABCB11 gene (BSEP protein). PFIC3 is due to mutations in the ABCB4 gene (MDR3 protein). New variants of PFIC have been recently described: PFIC4, resulting from a loss of function of tight junction protein 2 (TJP2), and PFIC5, caused by mutations in the NR1H4 gene. Moreover, mutations in the MYO5B gene have been associated with PFIC as it has a vital role in the trafficking of BSEP protein and hepatocyte membrane polarization [4,5] Other genetic defects involved in cholestasis include a deficit of alpha-1-antitrypsin (AATD) [6] or mutations in JAG1 that cause Alagille Syndrome (ALGS) type 1 or NOTCH2, leading to ALGS type 2 [7].

Therapeutic options for cholestasis are scarce and currently involve pharmacological approaches such as treatment with ursodeoxycholic acid or obeticholic acid or liver transplantation for the more severe cases [8] Currently, the practice guidelines for cholestasis treatment point out that accurate diagnosis and evaluation of parameters such as levels of ALP, AST/ALT, GGT, or liver stiffness, among others, as well as the cause of the cholestasis, are essential requirements. However, a detailed description of the molecular background of the disease would allow us to elaborate a more efficient precision medicine-based approach. The ultimate goal would be to discriminate patients suffering from different types of cholestasis to elaborate tailor-made treatments [9].

The recent developments in liquid chromatography coupled with tandem mass spectrometry (LC-MS) allow for the simultaneous identification and quantification of thousands of proteins in a biological specimen. Although the intrinsic nature of mass spectrometry-based proteomics is not quantitative, different approaches can be used to determine the abundance of proteins. Among them are data-dependent acquisition label-free quantification (DDA LFQ), data-independent acquisition label-free quantification (DIA LFQ), isobaric labeling (e.g., TMT, ITRAQ) [10], or targeted proteomics, where a predefined list of proteins and proteotypic peptides to be monitored is required [11]. Quantitative proteomics analyses focused on protein abundance and posttranslational modifications (PTMs) measurements reveal cell signaling and molecular mechanisms, which are central for disease progression and propel the discovery of biomarkers with promising applications for patient management [12].

Machine learning has been widely used in biology to analyze complex data generated in high-density studies. With the advent of high-throughput proteomics technologies, there has been a growing interest in modeling proteomics datasets with machine learning methods [13,14]. The final goal is to discriminate across different classes of individuals by constructing classifiers that facilitate an objective definition of specific phenotypes. This approach has been successfully used to define a protein profile characteristic of early pulmonary arterial hypertension in systemic sclerosis that allowed the classification of patients with high accuracy (81.1%), sensitivity (77.3%), and specificity (86.5%) [15].

In this study, we examined the liver proteome of 40 subjects with etiologically different cholestasis (biliary atresia, BA; Alagille Syndrome, ALGS; alph1 1 antitrypsin disease, AATD; progressive familial intrahepatic cholestasis, PFIC) to explain the molecular mechanisms underlying its pathogenesis. Moreover, we ranked the identified proteins according to their importance to classify the cases under study using iterative random forest analysis. Then, based on the calculated importance, machine learning algorithms were used for feature selection of the variables, resulting in a panel of 20 proteins that classified the cases according to the etiological groups with 91% accuracy.

## 2. Results and Discussion

### 2.1. Proteomics Analysis

**Figure 1:**
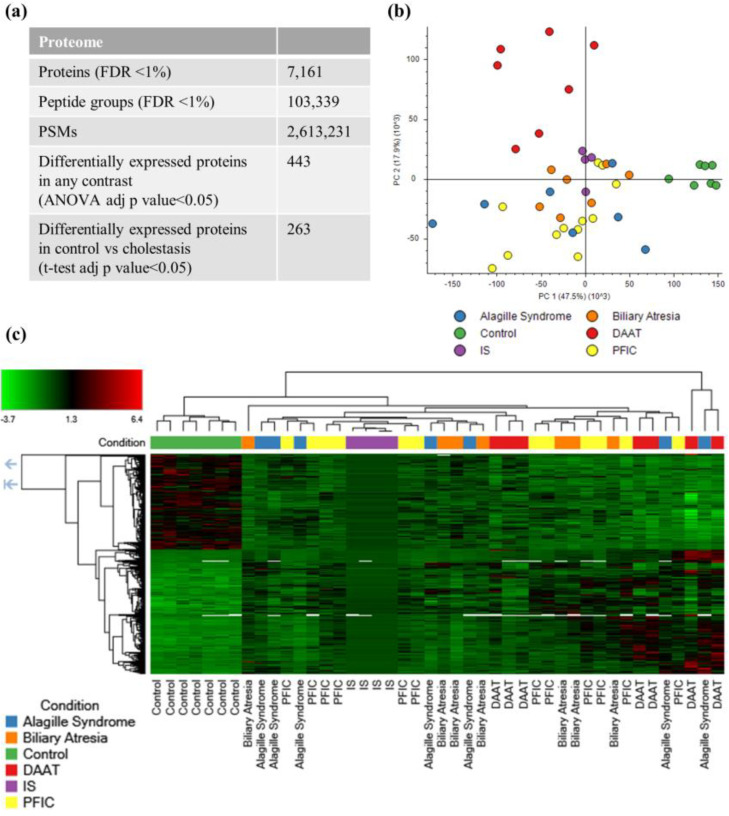
Quantitative proteomics results. (**a**) Table showing a summary of the number of PSMs, peptides, and proteins identified and differentially expressed in cholestasis (FDR < 1%). (**b**) PCA analysis of a proteomics dataset showing the segregation of cholestasis and control samples. IS corresponds to the internal standard containing equal amounts of each sample (ANOVA adj. *p*-value < 0.05). (**c**) Heatmap and clustering of differentially expressed proteins in cholestasis (ANOVA adj. *p*-value < 0.05).

Cholestasis is a liver disease condition that may result from different factors. Aiming to dig deeper into the molecular background of cholestasis, the proteome of liver samples from control and cholestatic patients was compared. Overall (Figure 1a), we identified 7161 protein groups with an FDR < 1% cut-off (Supplementary Table S2), of which 443 differential proteins were detected by ANOVA testing (Supplementary Table S3). However, as observed in the PCA and heatmap (Figure 1b,c), the discriminatory power of the differential proteins according to the ANOVA test was limited. It is worth mentioning that the internal standard appeared in the central region of the PCA plot, as expected from its composition (a mixture of all analyzed proteomes), which supports the robustness of the analysis (Figure 1b). While cases were clearly separated from the control group in the PCA analysis, only DAAT samples were clustered together and could be differentiated from other etiologies. At the same time, PFIC, ALGS, and biliary atresia were not segregated. Similar results were obtained when differential proteins were represented as a heatmap (Figure 1c) where only control samples were clustered together and displayed a typical protein pattern, different from cholestasis cases. These results suggested the close molecular phenotype of cholestasis subtypes and that despite the detection of statistically significant differences across them, further analysis was needed to improve the stratification of patients based on proteomics profiles.

**Figure 2:**
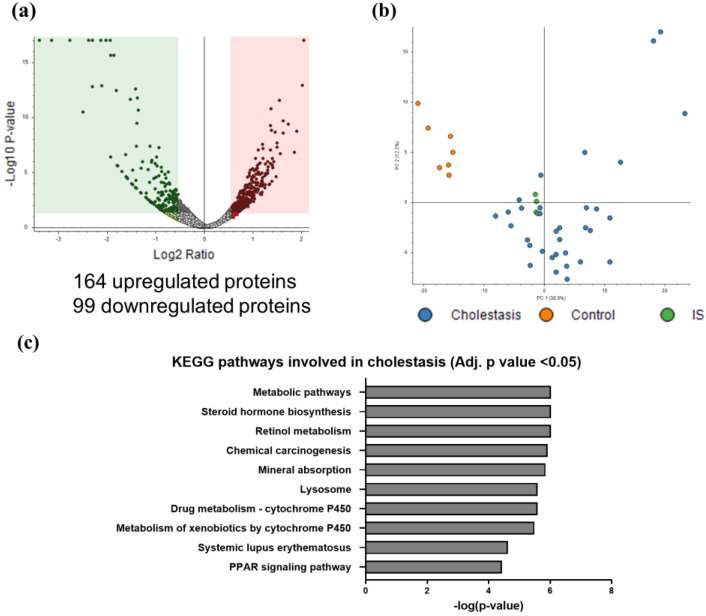
Functional analysis of the cholestatic proteome. (**a**) Volcano plot of differential proteins when control and cholestasis samples were compared (*t*-test adj. *p*-value < 0.05). (**b**) PCA analysis of proteomics dataset showing the segregation of cholestasis and control samples. IS corresponds to the internal standard containing equal amounts of each sample (*t*-test adj. *p*-value < 0.05). (**c**) Cellular processes altered in cholestasis using the list of differentially expressed proteins in cholestasis regardless of the etiology (KEGG pathways enrichment using KOBAS tool, adj. *p*-value < 0.05).

To further understand the functional implications of the proteome changes in cholestasis, control and cholestasis groups (regardless of the etiology) were compared, resulting in a list of 263 differentially expressed proteins, (*t*-test adj. *p*-value < 0.05) (Figure 2a; Supplementary Table S4). Both groups were well discriminated by PCA analysis (Figure 2b). Functional enrichment analysis (Figure 2c) revealed that the main cellular processes altered in cholestasis included metabolism (steroid hormone biosynthesis, drug metabolism by cytochrome P450, metabolism of xenobiotics by cytochrome P450, insulin signaling pathway, and retinol, pyruvate, and propanoate metabolism), chemical carcinogenesis, mineral absorption, lysosome, PPAR signaling pathway, phagosome, and immune processes, among others. Metabolic rewiring and inflammation appeared to be central components of liver injury progression associated with cholestasis and agree well with a previous study performed by our group focused specifically on PFIC3 [16].

### 2.2. Machine Learning Analysis

**Figure 3:**
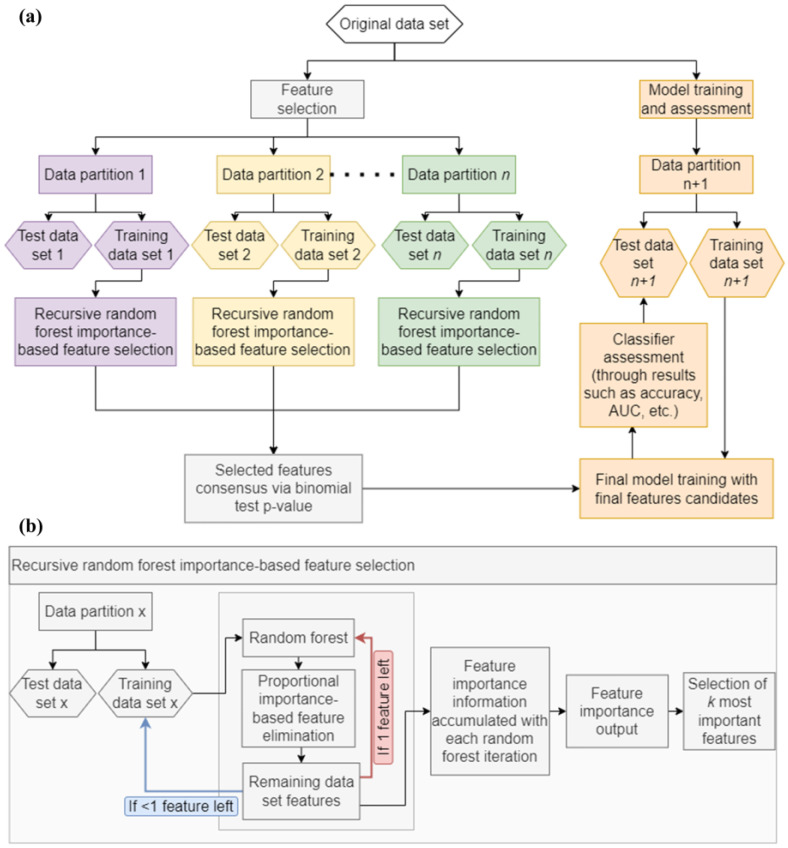
Machine learning analysis graphical workflow. (**a**) Workflow for iterative random forest-based feature selection and posterior model building and evaluation. The workflow involved dividing the original dataset into a 30–70% split for training and testing, ensuring an equal representation of samples from each class. We repeated this process 100 times. We conducted iterative random forest-based feature selection within each partition on the training set. We then used a binomial test *p*-value to select the top 20 features consistently appearing in the highest-ranked features across a significant portion of the data partitions. Using these selected features, we constructed a final model in a new dataset partition using various classifiers. We then assessed the performance of these classifiers (e.g., accuracy, area under the curve) using the test set from that partition. (**b**) Detailed view of recursive random forest importance-based feature selection. In the recursive random forest importance-based feature selection process, the training set was employed for each data partition to select features based on their importance, which was determined through multiple iterations of the random forest algorithm.

As mentioned above, although statistically significant differences were found between cholestasis subtypes, the stratification of etiologically different samples using classical dimension reduction methods such as PCA or hierarchical clustering as shown in the heatmap was limited (Figure 1a,b). This is a common finding arising from clinical proteomics studies in which sample heterogeneity and the similarity of molecular phenotypes, frequently prevent discrimination across disease subclasses and, therefore, compromise the efficient stratification of patients. We then wondered if state-of-the-art data processing algorithms may offer new opportunities to retrieve valuable information from complex proteomics datasets that allow better discrimination across cholestasis classes. To address this question, our proteomics dataset was analyzed with a machine learning-based pipeline consisting of a first feature selection method phase using iterative random forests followed by model training and final classifier assessment in the test set (Figure 3 and Supplementary Figure S2).

Feature selection methods are essential in identifying potential biomarkers from proteomics data. These methods help select important attributes by eliminating redundant or irrelevant data, which reduces the dataset’s size and increases the classifiers’ accuracy [17,18]. Here, we applied an algorithm for feature selection based on feature importance for sample classification calculated from the iterative random forest to analyze the proteomics data of cholestasis of different etiology and patients’ stratification [19,20,21]. Random forest is an algorithm based on decision trees, a classification model known for its interpretability but lacking predictive power [22]. These limitations can be circumvented by relying on the following principles: (a) a bootstrap-based cross-validation method [23]; (b) limiting the number of randomized variables per decision tree.

One of the problems in the field of machine learning is known as “*n* << *p*”, where “*n*” represents the number of samples and “*p*” means the number of variables. This problem is a constant in “omics” disciplines due to the typically small number of samples that comprise datasets and the value record of thousands of genes, transcripts, proteins, or metabolites that define each sample. Random forest can be used as a variable selection method by calculating importance and creating a classification model with the selected variables. In this case, the model may be overfitted to the variables selected from a particular dataset [24,25]. Thanks to the implicit bootstrap and its ability to handle many variables simultaneously, the random forest becomes an exceptional candidate for working with datasets with few samples and many variables to reduce overfitting. In 2006, Díaz-Uriarte and Álvarez de Andrés [20] used random forest iteratively to eliminate the least important variables in each classification round of the algorithm, gradually reducing the proportion of variables in each iteration. On the other hand, Boruta [19] facilitated variable selection through iterative random forest and played a role in addressing the “*n* << *p*” problem in classification tasks.

To perform all calculations based on randomization, a seed was set in the R environment to ensure the reproducibility of computations. For machine learning calculations (Figure 3), data were partitioned into training and test subsets comprising 70% (*n* = 29 samples) and 30% (*n* = 11 samples) of the data, respectively (data partition was performed maintaining the proportion of all classes). After that, the training dataset was subjected to the importance ranking algorithm. The algorithm was executed by establishing 30 feature elimination rounds, meaning the original dataset was regenerated 29 times. The variable ranking algorithm using random forest performed the following steps. (1) Random forest was first executed: the importance value of each variable in the dataset was calculated, and the variables were sorted by their importance according to a random forest. (2) The importance value of each variable was accumulated. (3) Random forest was executed for the second time: A dataset with double the size of the original dataset was generated. This dataset contained a randomized version of each original variable. The importance value of each variable was compared to one of its randomized versions. If the value associated with the original variable was higher, a value of 1 was assigned; otherwise, 0 was assigned. These binary values were accumulated into a binary vector assigned to each variable, which was used to calculate a *p*-value. (4) Features were iteratively eliminated from the training dataset through multiple rounds, considering a proportion of the least important. A value of 1 was assigned to the retained features, while a value of 0 was assigned to those eliminated in this and successive rounds. (5) When only one variable remained in the dataset, the original dataset was regenerated, and steps from 1 to 4 were repeated. (6) Variables were sorted in descending order of cumulative importance in a table. Then, *p*-values were calculated using a binomial test from the accumulated hits and non-hits (1 s and 0 s). The variable ranking algorithm sorted all proteins in the dataset according to their importance value. The top 20 variables were chosen from this list based on their highest importance.

Random forest and stepwise iterative importance-based feature elimination allowed us to obtain a robust subset of important features. By using many elimination steps and repeating the entire elimination process many times, evidence accumulated to confirm that the selected features are valuable in the classification processes. Nevertheless, and more importantly, when the number of samples was reduced, the results could be affected by the selected samples in the training dataset.

To reinforce the idea that a specific data partition did not bias the selected features, the whole process was iterated on different data partitions on training and test subsets. By choosing the “k” (20) most important features of each partition, the number of iterations a feature appeared among the most important ones could be calculated. Finally, a subsequent binomial test was used to assign a *p*-value to each feature designed as important in at least one partition.

In detail, the binomial test *p*-value was calculated as follows: the union set of all features that were among the most important at some partition is computed. After that, a number of hits were calculated for each feature, which equals the number of partitions a feature was selected as one of the 20 most important. Next, the hit probability was calculated by dividing the sum of the instances each feature was part of the 20 most important ones by the product of all proteins and the number of partitions. This hit probability, the number of hits, and the number of partitions were used to finally measure the *p*-value of each feature. From these results, a set of significant features using the conventional threshold of 0.05 was concluded.

In this work, 100 different data partitions were made. Overall, 308 proteins were classified as important in at least one partition. Ultimately, only 37 of these 308 proteins had a binomial test-adjusted *p*-value ≤ 0.05 (Supplementary Figure S1). The high number of proteins that at least appeared once among the top 20 important suggested there were false positive biomarker candidates; false positive biomarker candidates appear when only one data partition was performed, likely resulting from the restricted number of samples. To overcome this limitation, we preserved the 20 most significant proteins among the 37 significant proteins to build the machine learning classifiers.

**Figure 4:**
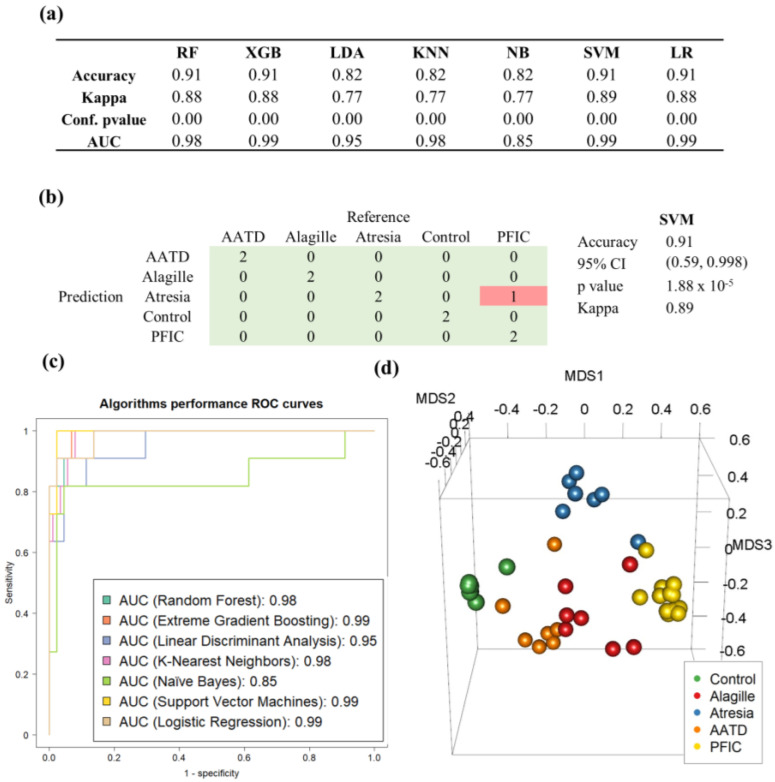
Machine learning analysis. (**a**) Performance of different algorithms: random forest, extreme gradient boosting, linear discriminant analysis, k-nearest neighbors, naïve Bayes, support vector machine, and logistic regression. (**b**) Confusion matrix of SVM algorithm; (**c**) ROC curves; (**d**) MDS (multidimensional scaling) 3D plot of samples.

These 20 proteins (Table 1) were used to train seven classification algorithms: random forest (RF), extreme gradient boosting (XGB), naïve Bayes (NB), k-nearest neighbors (KNN), support vector machine (SVM), logistic regression, and linear discriminant analysis (LDA) (Figure 4a). All classifiers were trained using leave-one-out cross-validation (LOOCV) as their cross-validation-associated method. All classification algorithms provided very high classification accuracy (>0.82) and area under the ROC curve values (>0.85) after testing them with the testing dataset (Figure 4c).

The SVM algorithm achieved the highest kappa coefficient, corresponding to only one misclassified sample in the test dataset. This misclassified sample was a PFIC case that was grouped with the biliary atresia cases (Figure 4b).

**Figure 5:**
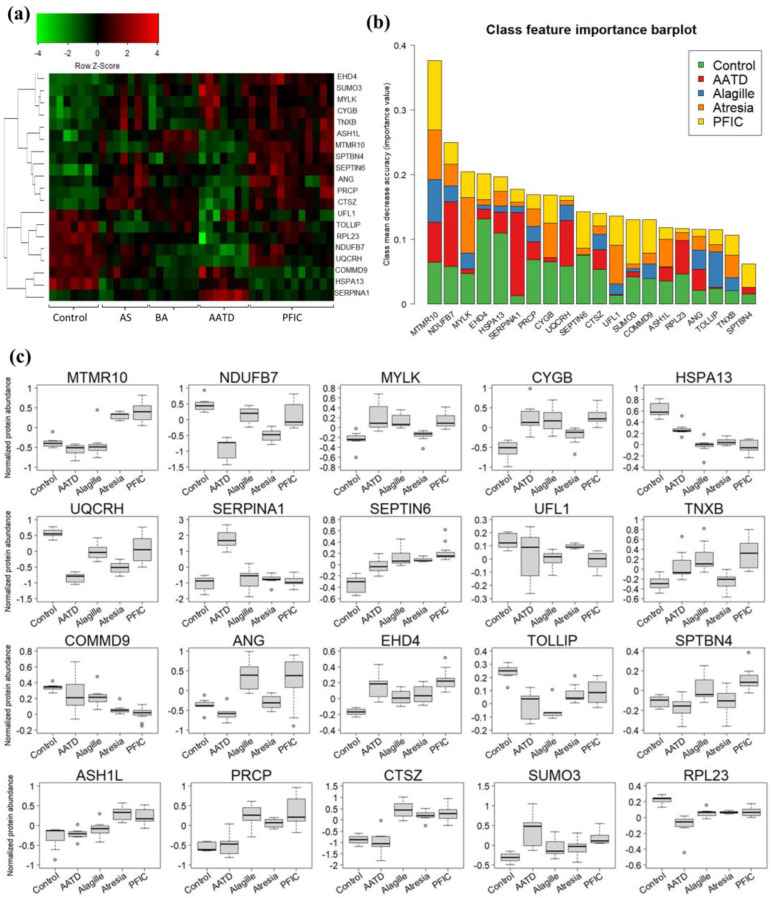
Classification of cholestasis of different etiologies using the described machine learning pipeline. (**a**) Heatmap representation of cholestasis subtypes clustering using the 20 proteins panel selected by the algorithm. (**b**) Contribution of each protein to the classification of each cholestasis group based on their estimated importance. (**c**) Boxplots representing the abundance values of each protein from the classifier in the different cholestasis groups.

Consistently, the MDS (multidimensional scaling) plot showed a clear group segregation except for one ALGS sample (Figure 4d). Next, a random forest final model was generated using the previously selected 20 variables and considering all samples together to calculate the variable importance associated with each class (Figure 5a,b) and a statistical-descriptive representation such as boxplots (Figure 5c); a straightforward interpretation of the model was achieved in terms of the overall relevance of variables in ensuring correct general classification, as well as the relevance of variables considering their importance classifying each specific class, and the direction in which each variable is important based on the relative values of samples from each class compared to the rest. As no samples were included in this study, the performance of the model for other genetic disorders leading to cholestasis remains to be investigated.

It is worth mentioning that most of the proteins from the panel (Table 1) are involved in cytoskeleton organization, metabolism and lysosomal activity, or inflammation, which are cellular processes identified as dysregulated in cholestasis in the functional analysis of the proteome (Figure 2a). Some of the proteins involved in cytoskeleton organization are myotubularin-related protein 10 (MTMR10), myosin light chain kinase (MYLK), spectrin beta, non-erythrocytic 4 (SPTBN4), or SEPTIN6. Maintaining the cytoskeleton organization and the apicobasal configuration of the hepatocytes is essential to preserve their function, including bile secretion. Loss of hepatocyte polarization leads to redistribution of bile transporters and results in pathological processes connected with bile retention in the liver, like cholestasis [26,27]. According to the Human Protein Atlas, EHD4 controls membrane reorganization, and TNXB mediates cell interactions and extracellular matrix interactions. It also accelerates collagen fibril formation and may play a role in supporting the growth of epithelial tumors. NDUFB7 (NADH:ubiquinone oxidoreductase subunit B7) is a protein involved in the mitochondrial membrane respiratory chain NADH dehydrogenase (Complex I), and UQCRH (Ubiquinol-cytochrome c reductase hinge protein) is a component of the ubiquinol-cytochrome c oxidoreductase. This multisubunit transmembrane complex is part of the mitochondrial electron transport chain, which drives oxidative phosphorylation. Altered mitochondrial functions have been observed in chronic liver diseases, including alcohol-induced liver disease, nonalcoholic fatty liver disease, viral hepatitis, liver regeneration [28], and primary and secondary cholestasis. Major changes included impairment of the electron transport chain and/or oxidative phosphorylation, leading to decreased oxidative metabolism of various substrates and decreased ATP synthesis [29]. Alterations in mitochondrial function can lead to oxidative stress-induced damage. Related to this, cytoglobin (CYBG) has proved to be protective against oxidative stress [30].

Moreover, lysosomal function has been documented to be disturbed in cholestasis models [31]. Following this, three proteins from our panel are involved in lysosomal autophagy activity (LAMP2, PRCP, and CTSZ). Concomitantly, other panel proteins participate in protein degradation through the proteasome pathway. HSPA13, UFL1, SUMO3, and COMMD9 are proteins related to ubiquitination and proteasome, suggesting the importance of this pathway as a potential source for biomarkers in cholestasis. Alterations in the E3 ubiquitin ligases have already been described in PFICII [32] COMMD9 participates in E3 ubiquitin ligase activity regulation and in cholesterol homeostasis and NF-κB pathway activation.

Interestingly, TOLLIP is a component of the signaling pathway of toll-like receptors, connecting the ubiquitin pathway to autophagy by functioning as a ubiquitin-ATG8 family adapter, thus mediating autophagic clearance of ubiquitin conjugates [33] Toll-like receptors are highly expressed in immune system-related cells, which may indicate the infiltration of immune cells in the liver of the cholestasis cases here analyzed, indicating liver inflammation. In agreement with this hypothesis, MTMR10 is also expressed in the nucleus of immune system cells. ASH1L is a histone methyltransferase whose dysregulation may result from impaired S-Adenosylmethionine metabolism, a common feature of liver diseases [34] including cirrhosis, hepatocellular carcinoma [35] and PFIC3 [16]. Angiogenin (ANG) induces angiogenesis after binding to actin on the surface of endothelial cells and plays an essential role in cell growth and tumor progression. SERPINA1 (alpha-1-antitrypsin) is a serine protease inhibitor belonging to the serpin superfamily whose targets include elastase, plasmin, thrombin, trypsin, chymotrypsin, and plasminogen activator. In alpha-1-antitrypsin deficiency (AATD), SERPINA1 is not released to serum, accumulating in the liver as observed in the boxplot (Figure 5c). Consequently, this protein is especially relevant for classifying AATD samples (Figure 5b). Finally, cytoglobin activation in all cases could result from stellate cell activation as a response to control free radical production, which is associated with fibrogenesis, a common feature of all types of cholestasis [36] Most of the proteins included in the panel have been identified in plasma according to Pax-DB [37] (Supplementary Table S5), reinforcing their potential as targets for the development of clinical applications.

In conclusion, the combination of proteomics and machine learning analyses yielded a detailed molecular phenotype of cholestasis and a panel of 20 proteins that allowed the stratification of etiologically different cholestatic patients with 91% accuracy. This approach opens new horizons for developing diagnostic, prognostic, and therapeutic strategies according to personalized and precision medicine principles.

## 3. Materials and Methods

### 3.1. Biological Samples

Liver tissue fragments from the explanted livers of cholestasis patients (PFIC3 *n* = 12, AATD *n* = 7, biliary atresia *n* = 7, ALGS *n* = 7) and from the liver graft of living donors (N = 7) (Supplementary Table S1) were obtained at the time of liver transplantation and stored frozen at −80 °C in RNAlater solution (Invitrogen, Carlsbad, CA, USA). Control samples were obtained from liver specimens from the living donors of LDLT (Living Donor Liver Transplant) surgeries. Pathogenic mutations were documented by genetic analysis. Liver transplants were performed between 0 and 10 years old. All surgical procedures were conducted in Hospital Universitario La Paz, Madrid, Spain. Liver samples were kept at the institutional biobank. Written informed consent was obtained from the patient’s legal guardians and graft donors. Study protocols conformed to the principles stated in the Declaration of Helsinki and were approved by the Clinical Research Ethics Committee of Hospital Universitario La Paz (HULP PI-3252).

### 3.2. Proteomics Analysis: Sample Preparation and LC-MS/MS Conditions

For sample preparation, liver specimens were processed as described previously [16] First, hepatic tissue samples were mechanically disrupted with a Potter–Elvehjem homogenizer in lysis buffer containing 5% SDS (sodium dodecyl sulfate) (Sigma-Aldrich, ST Louis, MO, USA), 100 mM triethylammonium bicarbonate (Thermo Fisher Scientific, Waltham, MA, USA), and a protease/phosphatase inhibitor cocktail (Thermo Fisher Scientific). After 1 min sonication on the UP50H ultrasonic lab homogenizer (Hielscher Ultrasonics, Teltow, Germany), samples were centrifugated at 10,000× *g* for 5 min. Protein concentration was determined in the supernatant using the Pierce 660 nm Protein Assay, adding IDCR (Ion detergent compatibility reagent) (Thermo Fisher Scientific, Waltham, MA, USA). Supernatants were stored at −80 °C until use.

Before digestion, proteins were reduced and alkylated by adding 5 mM TCEP (tris(2-carboxyethyl) phosphine) and 10 mM chloroacetamide for 30 min at 60 °C. Protein digestion was performed in S-Trap filters (Protifi, Huntington, NY, USA). The amount of 80 µg of protein of each sample was diluted to 40 µL in 5% SDS, 1.2% phosphoric acid, and 354 µL of binding buffer (90% methanol; 100 mM TEAB) and then loaded to an S-Trap filter. Subsequently, the filter was washed 3 times with 150 μL of binding buffer. Finally, MS-grade trypsin (Thermo-Fisher Scientific) was added to each sample in a ratio of 1:20 (trypsin/protein) in 20 μL of 100 mM TEAB. Digestion was performed at 37 °C overnight in a wet chamber. After digestion, peptides were eluted by the addition of two stepwise buffers: first, 40 μL of 25 mM TEAB, and then, 40 μL of 80% acetonitrile and 0.2% formic acid in H_2_O, and finally, by a 2 min centrifugation at 3000× *g*. The peptide amount was determined using a Qubit 2.0 Fluorometer (Thermo Fisher Scientific). Eluted peptides were centrifuged to dryness in a Speed Vac concentrator (Eppendorf, Hamburg, Germany).

The amount of 25 μg of the resulting peptide mixtures was then labeled using TMT-11plex Isobaric Mass Tagging Kit (Thermo Scientific, Rockford, IL, USA) according to the manufacturer’s instructions in four parallel labeling reactions, as shown in Table 2. Labeled peptides of each reaction were pooled and used to analyze the proteome and the phosphoproteome.

To increase the protein coverage, high pH reversed-phase peptide prefractionation was performed with sulfonated divinylbenzene (CDS Empore™ SDB-RPS, Sigma-Aldrich) using a step gradient elution with increasing acetonitrile concentrations (0–60% ACN) in a 10 mM ammonium formate (NH_4_HCO_2_, pH 10.0), as described previously [16] Peptides were collected into 10 different fractions and dried and stored at −20 °C until LC-MS analysis.

For LC-MS analysis, peptides were first solubilized in a solution containing 2% acetonitrile (ACN) and 0.1% formic acid (FA). The peptide concentration was determined using Qubit 2.0 Fluorometric Quantitation (Thermo Fisher Scientific). For analysis, 1 µg of each fraction in 5 µL was injected into a nano-LC ESI-MS/MS (Liquid Chromatography Electrospray Ionization Tandem Mass Spectrometry). The system consisted of an Ultimate 3000 nano HPLC system (Thermo Fisher Scientific) coupled to an Orbitrap Exploris 240 (Thermo Fisher Scientific). Peptides were separated using a 50 cm × 75 μm Easy-spray PepMap C18 analytical column at 45 °C at a flow rate of 300 nL/min and a 120 min gradient ranging from 2% to 95% mobile phase B (mobile phase A: 0.1% FA; mobile phase B: 80% ACN in 0.1% FA).

For the mass spectrometry analysis, a data-dependent top-20 method was used for data acquisition in full scan positive mode, scanning 375 to 1200 m/z. Parameters were set as follows: survey scans resolution, 60,000 at m/z 200; normalized automatic gain control (AGC) target (%), 300; maximum injection time (IT), AUTO; fragmentation, top 20 most intense ions from each MS1 scan via higher-energy collisional dissociation (HCD); resolution for HCD spectra, 45,000 at m/z 100; AGC target, 50; maximum ion injection time, AUTO; isolation window of precursors, 0.7 m/z; exclusion duration, 45 s; HCD collision energy, 30. Precursor ions with single, unassigned, or six and higher charge states from fragmentation selection were excluded.

### 3.3. Data Analysis: Proteomics Searches, Statistical and Functional Analysis

Thermo raw files were processed using Proteome Discoverer (PD) version 2.5 (Thermo Fisher Scientific). A workflow combining four search engines was used for PSM: Mascot (v2.7.0), MsAmanda (v2.0), MsFragger (v3.1.1), and Sequest HT (2.0.0.24). The database used was the human proteome available at Uniprot database (February 2021); a target/decoy database built from sequences in the human proteome at Uniprot Knowledgebase (9606rev_20210219) was used for the searches. Posttranslational modifications for the searches were set as follows: dynamic modifications for pyrrolidone from Q (−17.027 Da) and oxidation of methionine residues (+15.9949 Da) and static modification for TMT reagents (+229.163 Da) on lysine and N-term of the peptide, as well as carbamidomethyl (+57.021 Da) on cysteine. Trypsin cleavage was configured as 2 missed cleavages maximum. The peptide precursor mass tolerance was 10 ppm, and MS/MS tolerance was 0.02 Da. The false discovery rate (FDR) for proteins, peptides, and peptide spectral matches (PSMs) peptides was set at 1%. The quantification values for proteins were calculated using the abundance of total peptide based on the reporter ions. Protein abundances were normalized using the internal standard of each experiment, and the statistical significance of the differential abundances across conditions was determined according to adjusted *p*-values of a *t*-test/ANOVA. A list of differentially expressed proteins between control and cholestasis conditions was used for the functional analysis using KOBAS KEGG pathway enrichment [38].

### 3.4. Machine Learning Analysis

All calculations performed to generate machine learning models were conducted using R version 4.1.3. From the original dataset consisting of 7161 proteins, contaminants and proteins with missing values were removed, resulting in a final set of 5484 proteins. The original protein abundance data were first transformed using the base 2 logarithm. Then, batch effects were eliminated by standardizing abundance values with the corresponding internal standard reference sample. Lastly, data were normalized using median normalization with the “proBatch” [39] package version 1.10.0 in R.

The following libraries were used for the various calculations: “randomForest” [40] library version 4.7-1.1; the “caret” [41] library version 6.0-93 to create the machine learning models; the “xgboost” [42] library version 1.6.0.1 to build extreme gradient boosting models; the “class” [43] package version 7.3-20 to generate the k-nearest neighbors (KNN) model; the “klaR” [44] library version 1.7–1 to create the naive Bayes models; the “MASS” library version 7.3-58.1 to generate linear discriminant analysis (LDA) model; the “glmnet” library version 4.1-6 to create the logistic regression classifiers [45]; the “e1071” library version 1.7-13 to build the support vector machine classifiers [46]; and the “verification” [47] package version 1.42 to generate receiver operating characteristic (ROC) curves. The ROC curves were based on the probability values assigned to each class in the form of a vector, which were then input into the “roc.plot” function.

## Figures and Tables

**Table 1:** Panel of 20 proteins for classifying cholestasis of different etiology. UniProt accession, protein description, gene name, and *p*-value determined by the algorithm.

Uniprot Accession	Protein Description	Gene ID	*p*-Value
Q9NXD2	Myotubularin-related protein 10	MTMR10	<0.0001
P17568	NADH dehydrogenase [ubiquinone] 1 beta subcomplex subunit 7	NDUFB7	<0.0001
Q15746	Myosin light chain kinase, smooth muscle	MYLK	<0.0001
Q8WWM9	Cytoglobin	CYGB	<0.0001
P48723	Heat shock 70 kDa protein 13	HSPA13	<0.0001
P07919	Cytochrome b-c1 complex subunit 6, mitochondrial	UQCRH	<0.0001
P01009	Alpha-1-antitrypsin	SERPINA1	<0.0001
Q14141	Septin-6	SEPTIN6	<0.0001
O94874	E3 UFM1-protein ligase 1	UFL1	<0.0001
P22105	Tenascin-X	TNXB	<0.0001
Q9P000	COMM domain-containing protein 9	COMMD9	<0.0001
P03950	Angiogenin	ANG	<0.0001
Q9H223	EH domain-containing protein 4	EHD4	<0.0001
Q9H0E2	Toll-interacting protein	TOLLIP	<0.0001
Q9H254	Spectrin beta chain, non-erythrocytic 4	SPTBN4	<0.0001
Q9NR48	Histone-lysine N-methyltransferase ASH1L	ASH1L	<0.0001
P42785	Lysosomal Pro-X carboxypeptidase	PRCP	<0.0001
Q9UBR2	Cathepsin Z	CTSZ	<0.0001
P55854	Small ubiquitin-related modifier 3	SUMO3	<0.0001
P62829	60S ribosomal protein L23	RPL23	<0.0001

**Table 2:** TMT labeling of cholestasis liver samples.

Tag/ TMT Experiment	1	2	3	4
126	Control	PFIC 3	PFIC 2	ALGS
127N	PFIC 3	Biliary atresia	ALGS	AATD
127C	Biliary atresia	PFIC 3	AATD	PFIC 1
128N	ALGS	AATD	Control	PFIC 4
128C	PFIC 3	Control	PFIC 2	Biliary atresia
129N	Control	PFIC 2	Biliary atresia	ALGS
129C	PFIC 3	Biliary atresia	ALGS	AATD
130N	Biliary atresia	ALGS	AATD	Control
130C	ALGS	AATD	Control	PFIC 4
131	AATD	Control	PFIC 1	Biliary atresia
131C	pool	pool	pool	pool

## Data Availability

All proteomics data will be available in the PRIDE repository upon accession to the project: PXD047823

## Supplementary Materials

The following supporting information can be downloaded at: https://www.mdpi.com/article/10.3390/ijms25073684/s1.

## Abbreviations

AATD	Alpha-1 antitrypsin deficiency
ALGS	Alagille Syndrome
BA	Biliary atresia
DDA	Data-dependent acquisition
DIA	Data-independent acquisition
KNN	K-nearest neighbors
LC-MS	Liquid chromatography coupled to mass spectrometry
NB	Naïve Bayes
LDA	Linear Discriminant Analysis
LFQ	Label-free quantification
PFIC	Progressive familial intrahepatic cholestasis
RF	Random Forest
SVM	Support Vector Machines
XGB	Extreme Gradient Boosting
