# Supplementary material for: Discrimination of Etiologically Different Cholestasis by Modeling Proteomics Datasets
Source: Int J Mol Sci. 2024 Mar 26;25(7):3684. doi: 10.3390/ijms25073684 (PMC11011353; doi:10.3390/ijms25073684)
Supplement: Supplementary file 1 [file ijms-25-03684-s001.zip › Supplementary table S5_plasma abundance.png.pptx]

## Slide 1
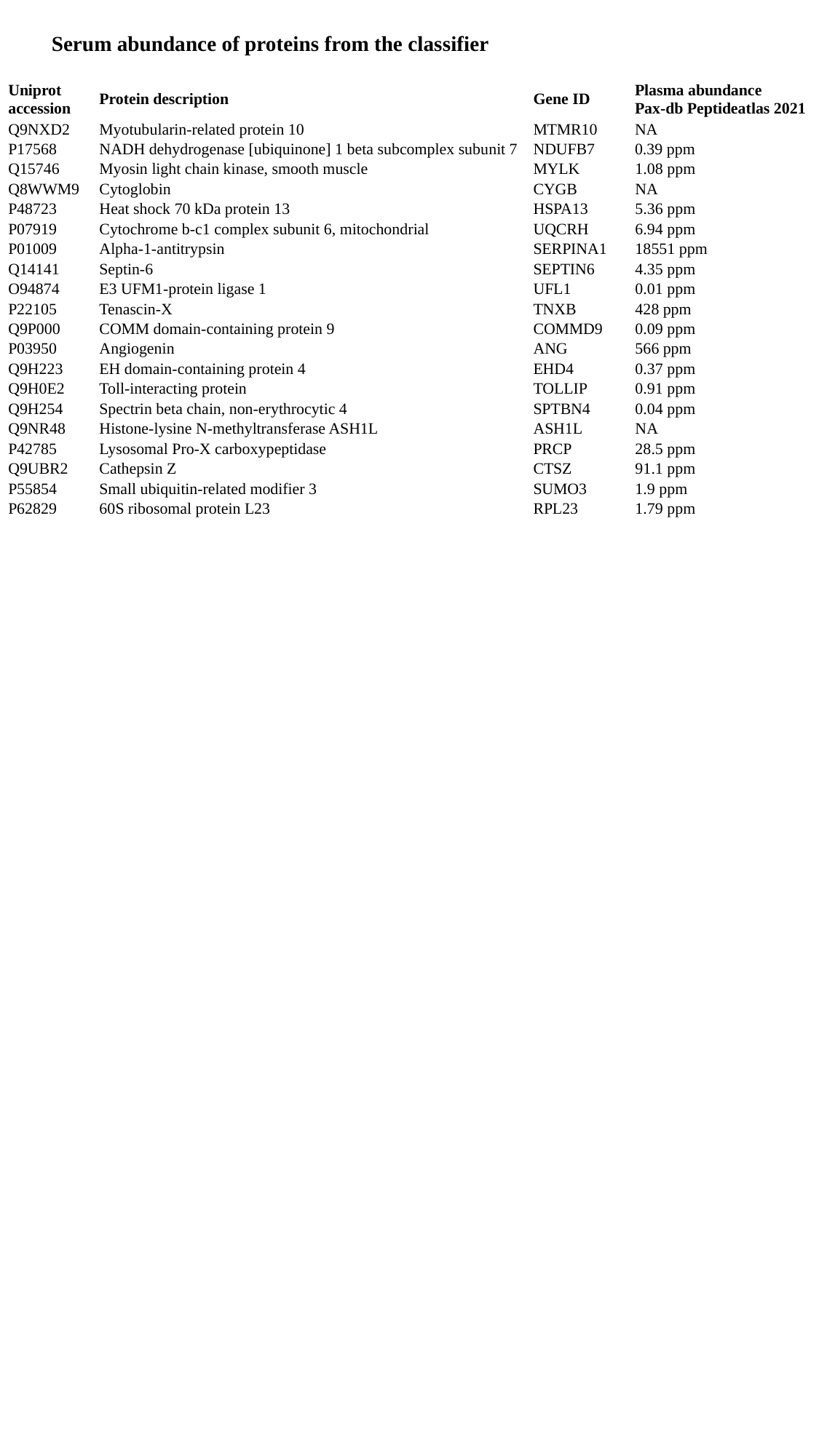

Serum abundance of proteins from the classifier
| Uniprot accession | Protein description | Gene ID | Plasma abundance Pax-db Peptideatlas 2021 |
| --- | --- | --- | --- |
| Q9NXD2 | Myotubularin-related protein 10 | MTMR10 | NA |
| P17568 | NADH dehydrogenase [ubiquinone] 1 beta subcomplex subunit 7 | NDUFB7 | 0.39 ppm |
| Q15746 | Myosin light chain kinase, smooth muscle | MYLK | 1.08 ppm |
| Q8WWM9 | Cytoglobin | CYGB | NA |
| P48723 | Heat shock 70 kDa protein 13 | HSPA13 | 5.36 ppm |
| P07919 | Cytochrome b-c1 complex subunit 6, mitochondrial | UQCRH | 6.94 ppm |
| P01009 | Alpha-1-antitrypsin | SERPINA1 | 18551 ppm |
| Q14141 | Septin-6 | SEPTIN6 | 4.35 ppm |
| O94874 | E3 UFM1-protein ligase 1 | UFL1 | 0.01 ppm |
| P22105 | Tenascin-X | TNXB | 428 ppm |
| Q9P000 | COMM domain-containing protein 9 | COMMD9 | 0.09 ppm |
| P03950 | Angiogenin | ANG | 566 ppm |
| Q9H223 | EH domain-containing protein 4 | EHD4 | 0.37 ppm |
| Q9H0E2 | Toll-interacting protein | TOLLIP | 0.91 ppm |
| Q9H254 | Spectrin beta chain, non-erythrocytic 4 | SPTBN4 | 0.04 ppm |
| Q9NR48 | Histone-lysine N-methyltransferase ASH1L | ASH1L | NA |
| P42785 | Lysosomal Pro-X carboxypeptidase | PRCP | 28.5 ppm |
| Q9UBR2 | Cathepsin Z | CTSZ | 91.1 ppm |
| P55854 | Small ubiquitin-related modifier 3 | SUMO3 | 1.9 ppm |
| P62829 | 60S ribosomal protein L23 | RPL23 | 1.79 ppm |
