# Supplementary figures and images for: Discrimination of Etiologically Different Cholestasis by Modeling Proteomics Datasets
Source: Int J Mol Sci. 2024 Mar 26;25(7):3684. doi: 10.3390/ijms25073684 (PMC11011353; doi:10.3390/ijms25073684)

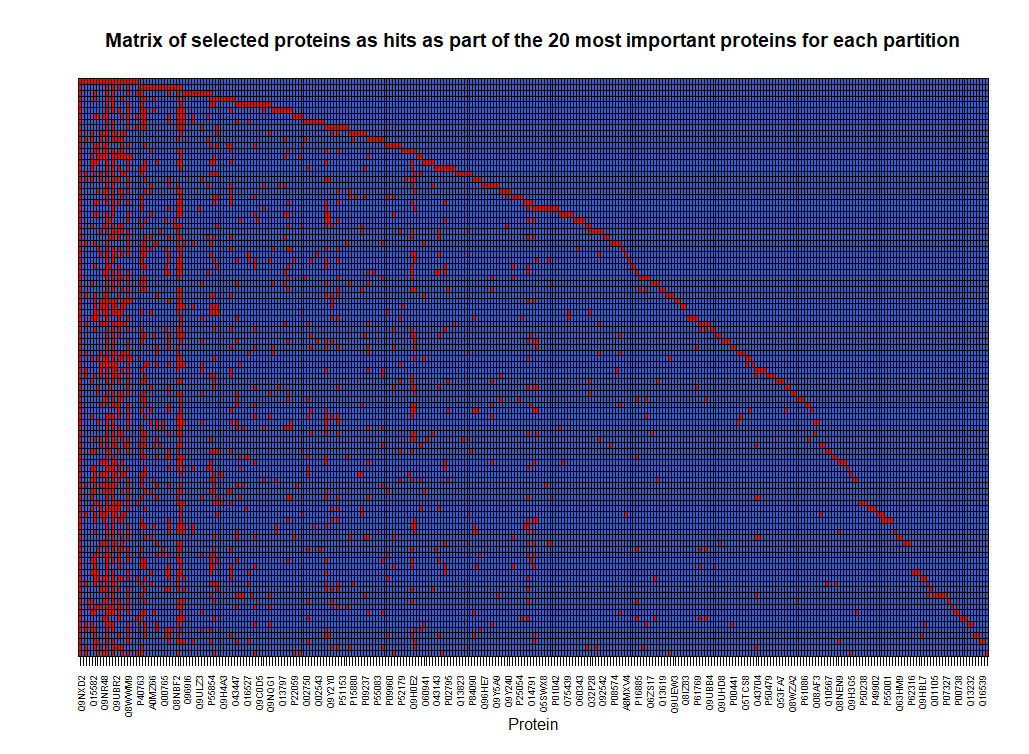

Supplement: Supplementary file 1 [file ijms-25-03684-s001.zip › Supplementary figure S1_top20_partitions.png]

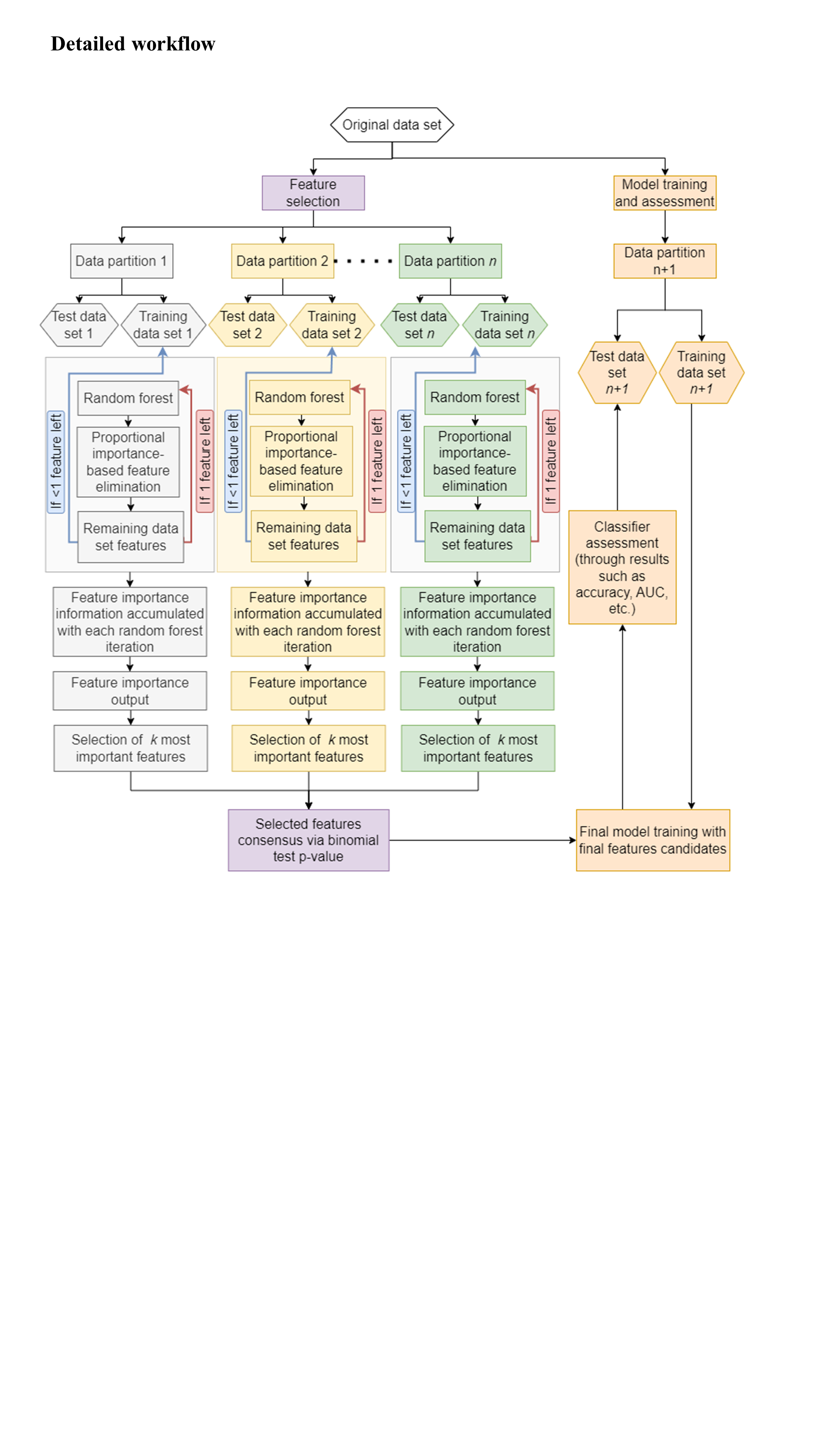

Supplement: Supplementary file 1 [file ijms-25-03684-s001.zip › Supplementary figure S2_Workflow detailed.PNG]
